# Inoperable Cancer

**Published:** 1901-10-26

**Authors:** 


### INOPERABLE CANCER.

There are few cases in which the line of treatment is more clearly laid down than in those examples of malignant disease in which there is a reasonable probability that by means of an operation the disease, together with its lymphatic prolongations, can be completely removed. If it can be removed the treatment is to remove it. Unfortunately for our patients and for our own peace of mind it is only now and again that the problem takes so simple a form. Cases are constantly being met with which are either so near the borderland that it is a serious question how far an operation will repay the risk which it involves or in which, worse still, it is obviously impossible to get rid of the disease by any justifiable operative measures. In those cases in which removal by operation is impossible one has then to inquire what other means there are of staying the disease or perchance of curing it. In his presidential address on the opening of the session of the West London Medical Society, Mr. Alfred Cooper dealt very fully with this important problem, passing in review the various measures of one kind and another which have been proposed of late years. The list is a long one and includes the following:—Inocculation with the streptococcus of erysipelas; subcutaneous injection with Coley's fluid; subcutaneous injection of anticancerous serum; oörphorectomy; thyroid feeding; lymph gland extract; treatment by X-rays and by Finsen's light; injection of various irritating substances and the production of aseptic suppuration; electricity, and a long string of drugs. Summing up the whole matter, Mr. Cooper thinks that we may arrive at the following conclusions:—1. That in cases of inoperable sarcoma, more especially the spindle-cell variety, the patient should have the option of Coley's fluid given to him, since a certain number of cases have been cured. 2. That in cases of inoperable cancer of the breast in women of about 40 years of age, in whom the menopause has not occurred, the operation of oöphorectomy should be proposed, and this treatment may be combined with thyroid feeding. 3. That in cases of inoperable rodent ulcer and in the superficial malignant ulcerations in other parts, the X-rays give a good hope of improvement. 4. That in cases where these other methods are declined or are inapplicable the internal administration of celandine is worthy of trial, and when the case appears quite hopeless morphia should be pushed without hesitation. 5. Finally, he would suggest that before trying any of these remedies the risk should be fully pointed out to the patient, that the faint hope that most of them afford should not be magnified, and that the discomfort of the treatment should be fully discussed; in fact, the surgeon should not do more than offer the treatment and leave the patient to accept or receive it.

